# Supplementary material for: The intrinsic time tracker: temporal context is embedded in entorhinal and hippocampal functional connectivity patterns
Source: Nat Commun. 2025 Oct 3;16:8817. doi: 10.1038/s41467-025-63633-6 (PMC12494713; doi:10.1038/s41467-025-63633-6)
Supplement: Supplementary file 1 — Supplementary Information [file 41467_2025_63633_MOESM1_ESM.pdf]

Supplementary Methods

Gray matter masks

We used FMRIB Automated Segmentation Tool (FAST) from the FSL software package to create gray matter masks from each subject's T1-weighted anatomical image[1]. Gray matter masks were then resampled to the functional resolution using FLIRT in FSL and thresholded at 50%. Voxels within the gray matter masks that fell within a given region of interest (ROI) were excluded from all analyses for that seed ROI[2].

Supplementary Results

Regional EC-HPC resting connectivity pattern drifts over time

Prior evidence shows that temporal information is represented within the medial temporal lobe (MTL[3–11]), consistent with our finding (reported in the main manuscript) that EC- and aHPC-whole brain resting connectivity patterns showed time-dependent changes (**Figure 2**).

Thus, these findings raise the possibility that intrinsic functional coupling patterns *between* EC and HPC—as indexed by resting functional connectivity—may likewise change according to objectively elapsed time. To test whether the similarity of functional connectivity patterns between EC and HPC changed over time, we next examined EC-HPC temporal drift scores. Following extant theoretical work suggesting that EC provides timing signals to HPC[12,13], we examined functional connectivity pattern changes across HPC voxels using EC as the seed region. We found a significant negative correlation between EC->HPC resting connectivity pattern similarity and the time interval elapsed between session pairs (Female: r = -0.158, p = 0.001; Male: r = -0.229, p < 0.001).

To ascertain the regional specificity of this finding, we performed the following two control analyses: EC-control ROI (EC as seed and a control region as a mask) and control ROI-HPC (control region as seed and HPC as a mask). We found that EC-HPC temporal drift scores were significantly stronger than temporal drift scores obtained in both control analyses (See **Supplementary Table 2**). Collectively, these results indicate that functional coupling patterns between EC and HPC systematically drift over time.

Individualized network parcellation analysis

To complement the group-level regional whole-brain functional connectivity analysis, we parcellated the cortex using a subject-specific, multi-session cortical parcellation method (for details, see Kong et al., 2019)[14,15]. The results from this individualized network parcellation analysis largely aligned with the group-level results for EC-network connectivity changes (**Supplementary Figure 4, Supplementary Table 5**). Specifically, using individualized network masks, we found that EC temporal drifts were primarily driven by the DMN-C (Female: $r = -0.209$, $p_{FDR} < 0.001$; Male: $r = -0.229$, $p_{FDR} < 0.001$), DMN-D (Female: $r = -0.163$, $p_{FDR} = 0.002$; Male: $r = -0.196$, $p_{FDR} < 0.001$), dorsal attention network A (DA-A) (Female: $r = -0.179$, $p_{FDR} = 0.001$; Male: $r = -0.2$, $p_{FDR} < 0.001$), dorsal attention network B (DA-B) (Female: $r = -0.174$, $p_{FDR} = 0.001$; Male: $r = -0.174$, $p_{FDR} < 0.001$), ventral attention network-A (VAN-A) (Female: $r = -0.156$, $p_{FDR} < 0.001$; Male: $r = -0.170$, $p_{FDR} < 0.001$), and Visual-B (Female: $r = -0.177$, $p_{FDR} < 0.001$; Male: $r = -0.278$, $p_{FDR} < 0.001$). These findings largely align with our original results, which had implicated networks DMN-C, DMN-D, and DA-A. Notably, these networks showed significantly stronger temporal drift than the somatomotor network in both subjects (EC-networks vs. somatomotor *ps* < 0.05), with the exception of the EC-DMN-D for the Female subject ($p = 0.088$).

However, when inspecting the individualized network-level results for aHPC, we noticed discrepancies between the two parcellation methods (**Supplementary Figure 4, Supplementary Table 6**). We found that aHPC temporal drifts were driven by the VAN-A (Female: $r = -0.302$, $p_{FDR} < 0.001$; Male: $r = -0.209$, $p_{FDR} < 0.001$) and the Visual network

(Visual-B) (Female: $r = -0.314$, $p_{FDR} < 0.001$; Male: $r = -0.235$, $p_{FDR} < 0.001$), similar to our original results, in which aHPC's connectivity change was driven by VAN-A and Visual-A. Note, however, these results were only significantly stronger than the control network in the Male subject (aHPC-VAN-A *vs*. control $p < 0.001$; aHPC-Visual-B *vs.* control $p < 0.001$), but not the Female subject (*ps* > 0.083). We also noticed some discrepancy in the individualized networks associated with aHPC relative to the group method—such as the emergence of the DA-B network for both subject (Female: $r = -0.274$, $p_{FDR} < 0.001$, aHPC-DA-B *vs*. control $p = 0.003$; Male: $r = -0.230$, $p_{FDR} < 0.001$, aHPC-DA-B *vs*. control $p = 0.027$) and the limbic network B (LIM-B) for the female subject only (Female: $r = -0.290$, $p_{FDR} < 0.001$, aHPC-LIM-B *vs*. control $p = 0.007$).

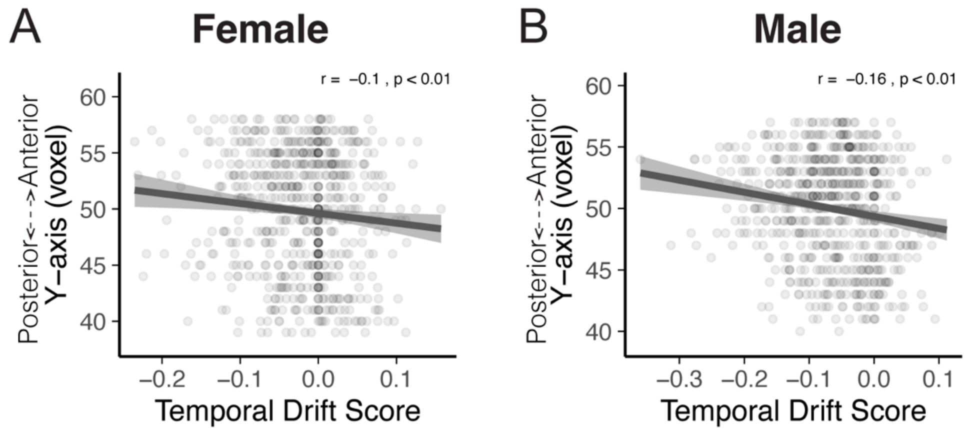

***Supplementary Figure 1*. Voxel-wise temporal drift scores were negatively correlated with the Y-axis coordinates in the hippocampus (A:** Female, **B**: Male).

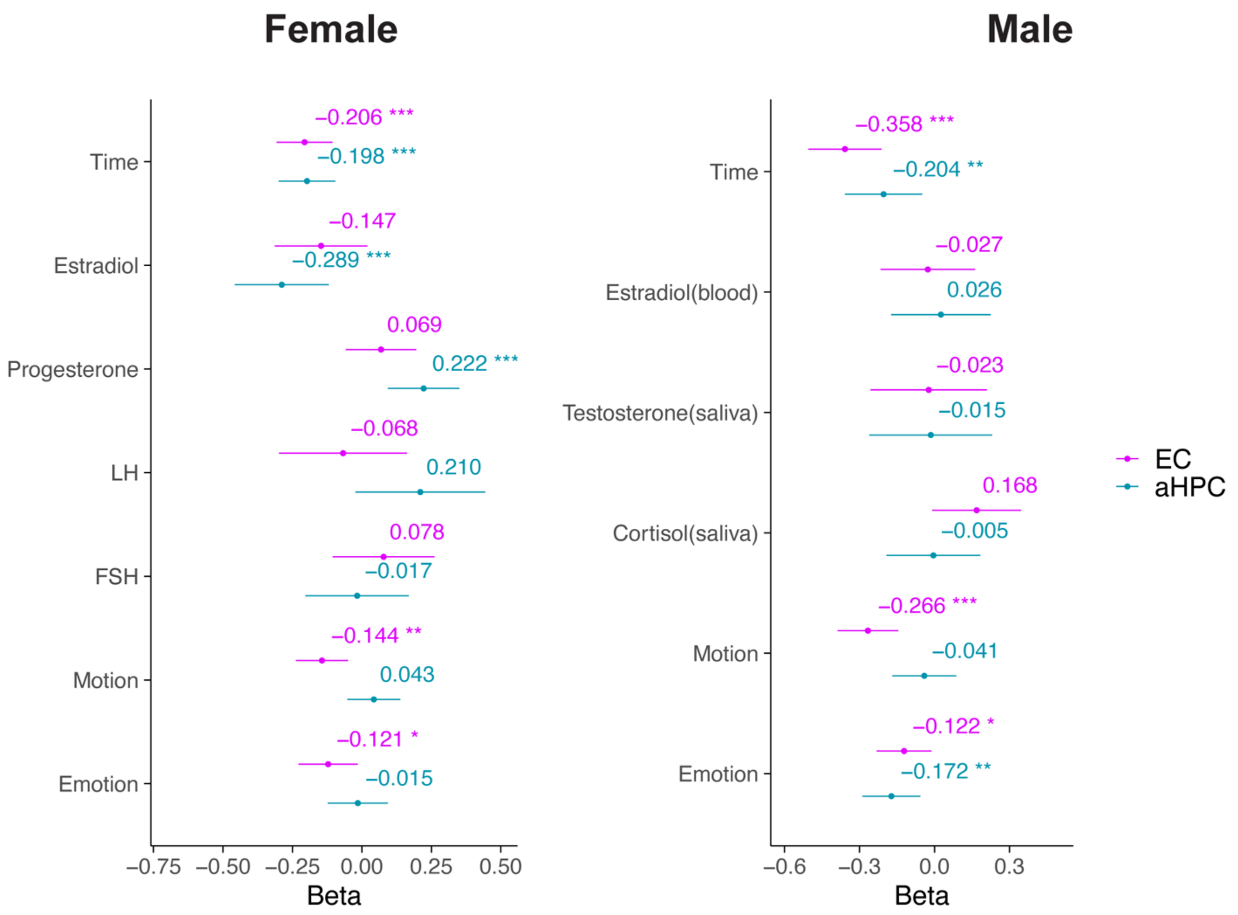

***Supplementary Figure 2*. The passage of time over a 30-day period is associated with decreased similarity in EC- and aHPC- whole-brain resting connectivity patterns after controlling for changes in hormones, emotion, and head motion.** Fixed-effect estimates (ß) were plotted with standard error (SE) bars for the female (left) and male (right) subjects. ß and SE are standardized for visualization purposes. Abbreviations: LH: luteinizing hormone, FSH: follicle stimulating hormone. * Denotes statistical significance at $p \leq 0.05$; ** Denotes statistical significance at $p \leq 0.01$; *** Denotes statistical significance at $p \leq 0.001$.

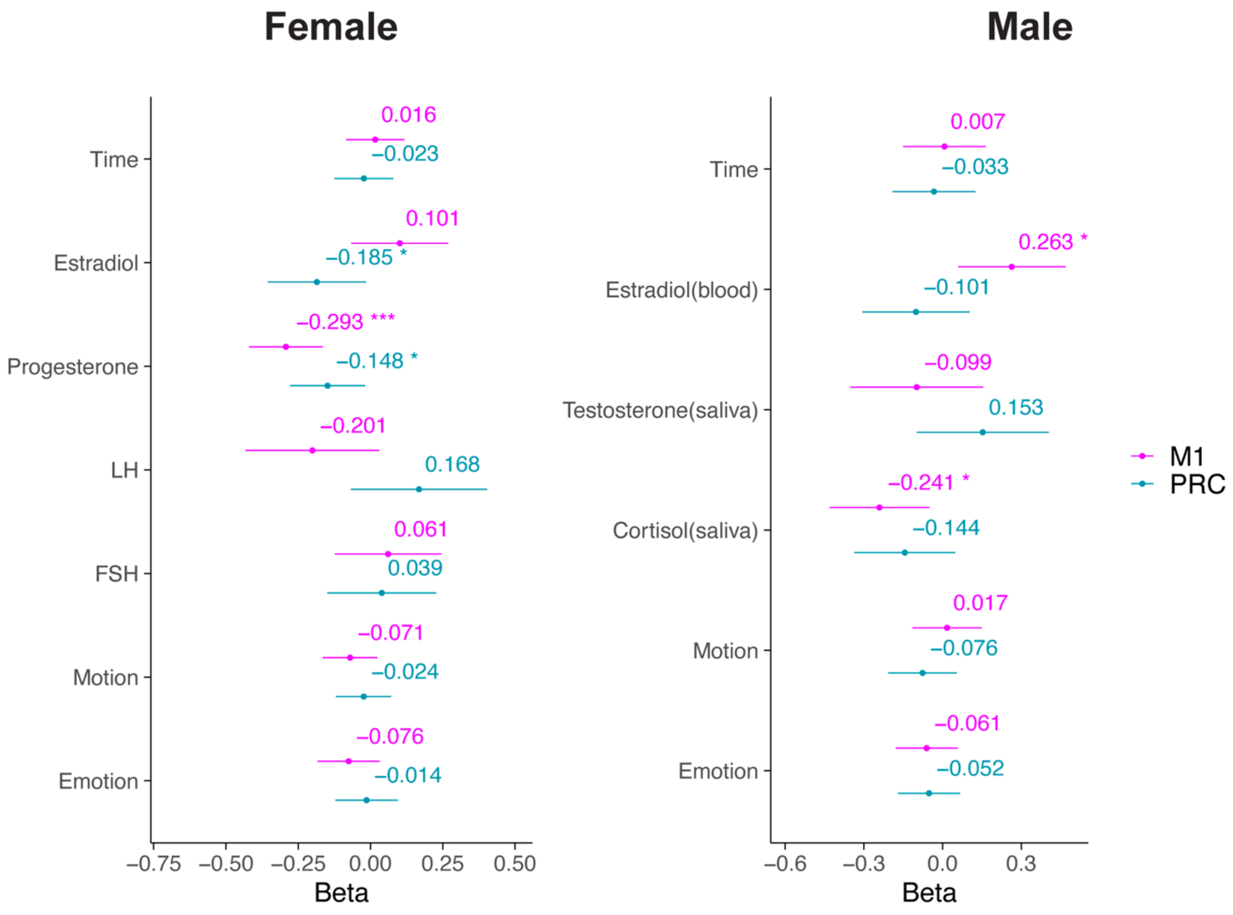

***Supplementary Figure 3*. Multiple regression of time-varying factors in control ROIs.** Only hormonal fluctuations (and not time interval) were associated with changes in similarity in M1- and PRC-whole-brain resting connectivity patterns in a simultaneous regression model that also included changes in emotion and head motion—in contrast to findings in EC and HPC (**Figure Supplementary Figure 2**). Fixed-effect estimates (ß) are plotted with standard error (SE) bars for the female (left) and male (right) subjects. ß and SE were standardized for visualization purposes. Abbreviations: LH: luteinizing hormone, FSH: follicle stimulating hormone. * Denotes statistical significance at $p \leq 0.05$;

** Denotes statistical significance at $p \leq 0.01$; *** Denotes statistical significance at $p \leq 0.001$.

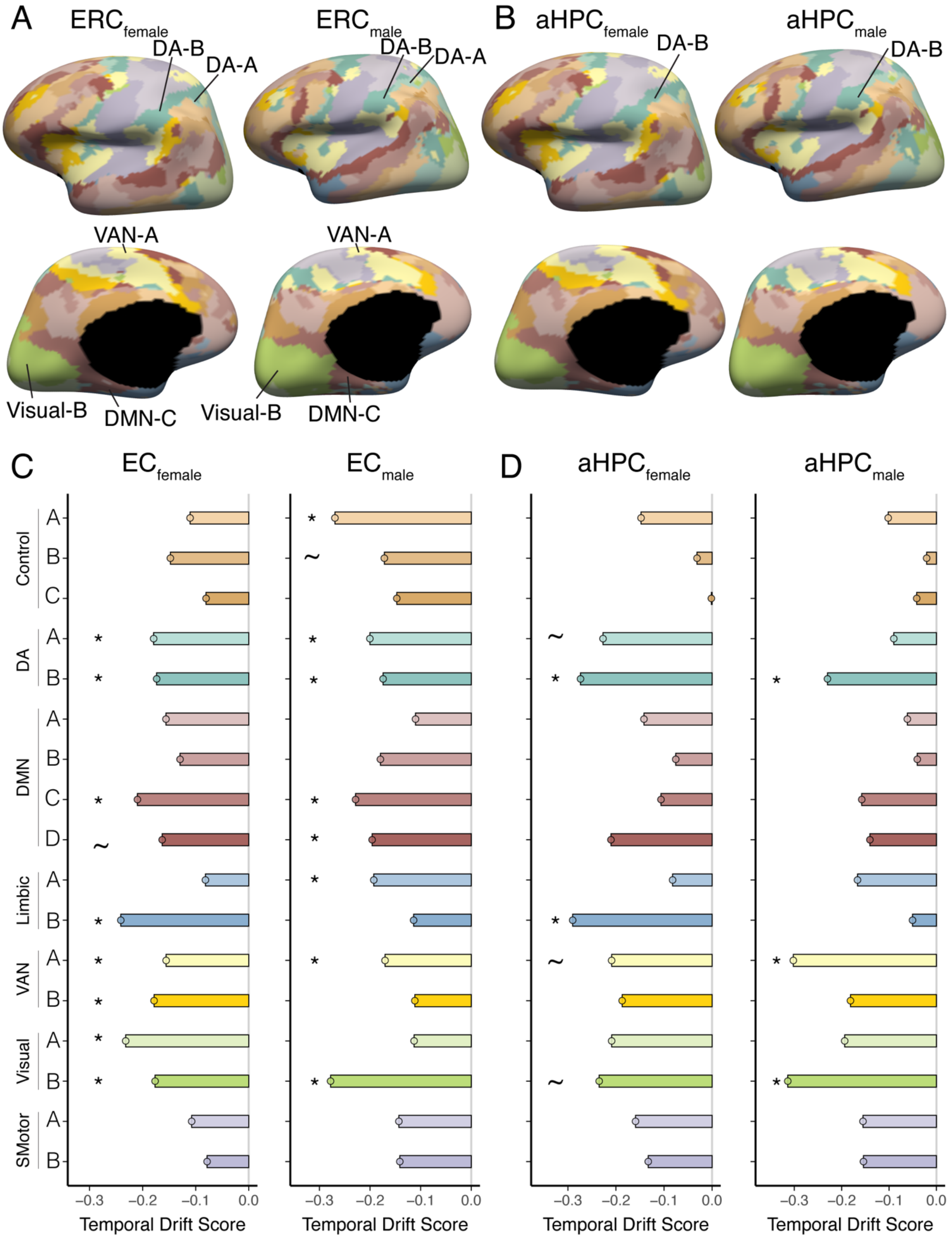
A
ERCfemale
DA-B
DA-A
ERCmale
DA-B
DA-A
B
aHPCfemale
DA-B
aHPCmale
DA-B
VAN-A
VAN-A
Visual-B
DMN-C
Visual-B
DMN-C
C
ECfemale
ECmale
D
aHPCfemale
aHPCmale
Control
DA
DMN
Limbic
VAN
Visual
SMotor
Temporal Drift Score
−0.3
−0.2
−0.1
0.0

Supplementary Figure 4. **Individualized network analysis: EC and aHPC time-dependent pattern changes were driven by specific networks.** Seventeen individually parcellated brain networks[15] are shown on a surface template (color-coded by network) for each participant. The primary networks that drove time-related resting connectivity pattern changes in the **(A)** EC and **(B)** aHPC—using the individualized network parcellation approach—are highlighted. Bar plots show temporal drift scores for **(C)** EC and the individualized Yeo-17 large-scale networks (Female: left, Male: right) and **(D)** aHPC and the individualized Yeo-17 large-scale networks (Female: left, Male: right). *Denotes significantly different temporal drift scores compared to the control somatomotor network in each subject. ~Denotes temporal drift scores were at trend level different than the control somatomotor network in each subject. Abbreviations: default mode network (DMN), dorsal attention network (DA), ventral attention network (VAN), somatomotor network (SMotor).

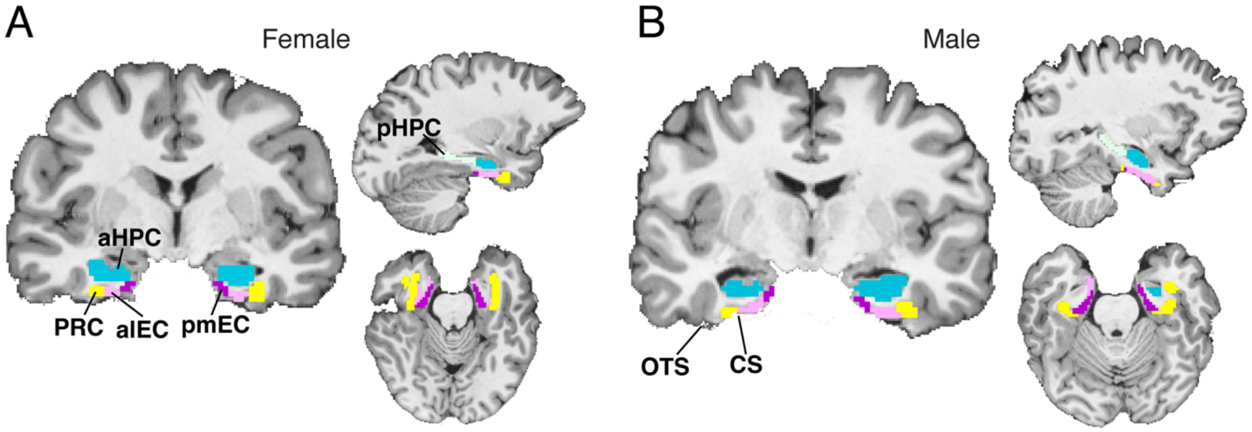

Supplementary Figure 5. **The medial temporal lobe masks are shown for the female (A) and male (B) participants.** Abbreviations: aHPC: anterior hippocampus, pHPC: posterior hippocampus, alEC: anterolateral entorhinal cortex, pmEC: posteromedial entorhinal cortex, PRC: perirhinal cortex, OTS: Occipital-temporal sulcus, CS: collateral sulcus.

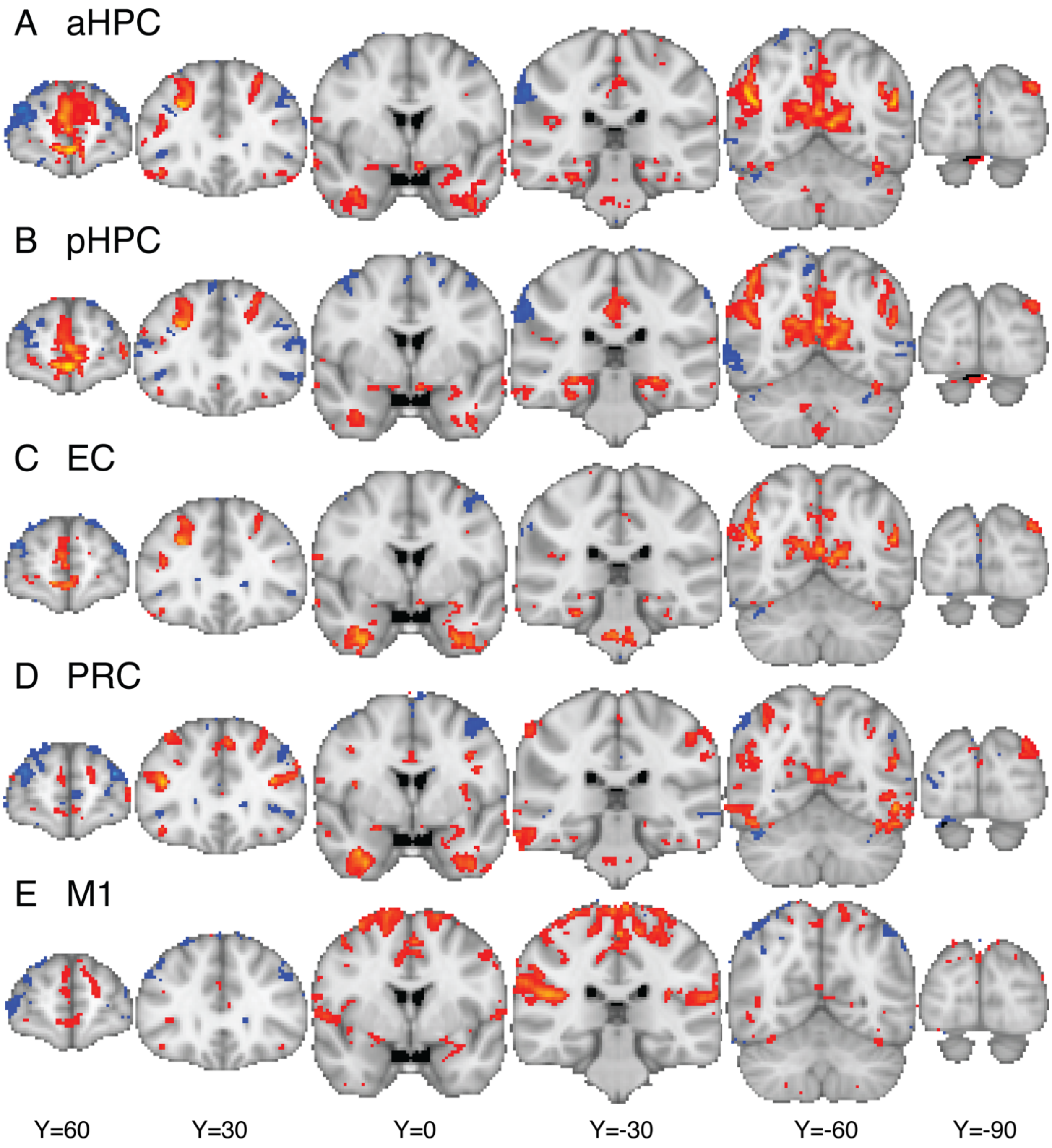

***Supplementary Figure 6*. Whole-brain functional connectivity maps for each ROI in the female subject. (A)** aHPC, **(B)** pHPC, **(C)** EC, **(D)** PRC, **(E)** M1.

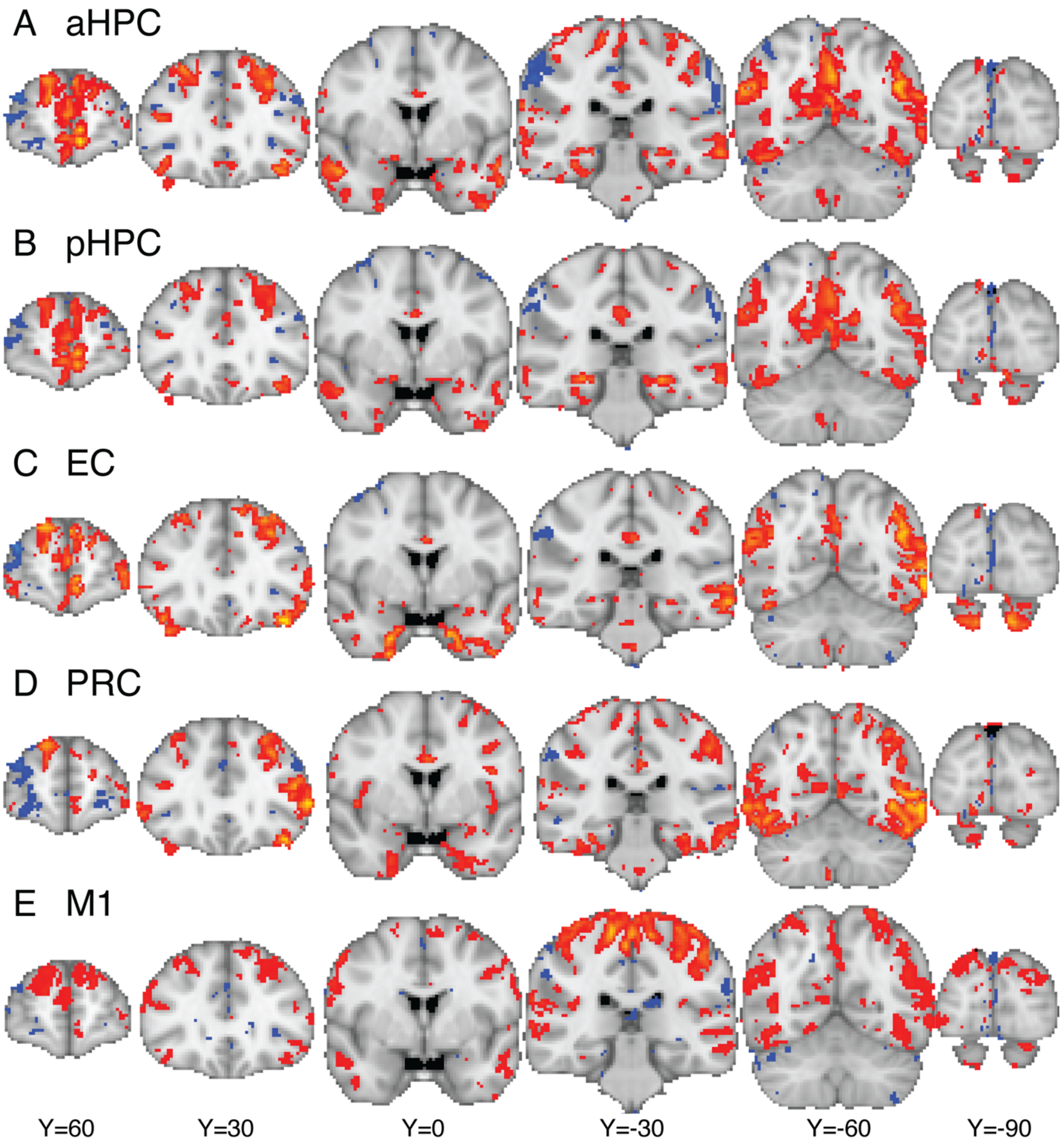

Supplementary Figure 7. **Whole-brain functional connectivity maps for each ROI in the male subject**. (**A)** aHPC, **(B)** pHPC, **(C)** EC, **(D)** PRC, **(E)** M1.

Supplementary Table 1: Temporal drift scores (Pearson's *r*) and comparison of correlation coefficients

	Female				Male			
	r	*p*	*vs.* M1, *p*	*vs.* PRC, *p*	r	*p*	*vs.* M1, *p*	*vs.* PRC, *p*
EC	**-0.206**	**< 0.001***	**0.010***	**0.016***	**-0.217**	**< 0.001***	**0.004***	**0.001***
aHPC	**-0.180**	**< 0.001***	**0.034***	**0.043***	**-0.153**	**< 0.001***	**0.051~**	**0.046***
HPC	**-0.187**	**< 0.001***	**0.026***	**0.025***	-0.146	< 0.001*	0.093~	0.076~
pHPC	-0.142	0.002*	0.098~	0.151	-0.123	< 0.001*	0.221	0.216
alEC	**-0.218**	**< 0.001***	**0.006***	**0.006***	**-0.179**	**< 0.001***	**0.027***	**0.017***
pmEC	-0.034	0.227	0.592	0.808	-0.111	0.001*	0.314	0.319
M1	-0.050	0.158	-	-	-0.087	0.006*	-	-
PRC	-0.089	0.032*	-	-	-0.091	0.007*	-	-

r: Temporal drift score calculated as Pearson's correlation coefficient; *p:* Permutation test *p*-value; *vs.* Control, *p*: Comparison of dependent correlation coefficients between EC and HPC ROI vs. control regions (M1 and PRC). * Denotes permutation test $p \leq 0.05$; ~ Denotes permutation test (at trend level) $p \leq 0.1$. Bolded text denotes temporal drift scores that are statistically significant (nonparametric permutation test) and specific (relative to the control regions, as indexed by the formal test of the difference between dependent correlation coefficients).

Supplementary Table 2: Temporal drift scores and comparison of correlation coefficients for EC-HPC

	**r**	***p***	***vs.* M1-HPC, *p***	***vs.* EC-M1, *p***	***vs.* PRC-HPC, *p***	***vs.* EC-PRC, *p***
EC-HPC (Female)	-0.158	<0.001*	0.010*	0.048*	0.022*	0.049*
EC-HPC (Male)	-0.229	<0.001*	0.002*	0.007*	0.001*	<0.001*

r: Temporal drift score calculated as Pearson's correlation coefficient; *p:* Permutation test *p*-value; *vs.*Control, *p*: Comparison of dependent correlation coefficients between EC and HPC ROI vs. control (M1 or PRC).

* Denotes statistical significance at *p* <= 0.05.

Supplementary Table 3: EC-network resting connectivity temporal drift scores

	Female			Male		
Network	r	p_{FDR}	*vs.* SMotor, *p*	r	p_{FDR}	*vs.* SMotor, *p*
Control_A	-0.132	0.005*	0.178	-0.227	< 0.001*	< 0.001*
Control_B	-0.144	0.003*	0.125	-0.177	< 0.001*	0.075~
Control_C	-0.055	0.131	0.715	-0.156	< 0.001*	0.283
**DA_A**	**-0.238**	**< 0.001***	**0.002***	**-0.275**	**< 0.001***	**< 0.001***
DA_B	-0.140	0.003*	0.083~	-0.248	< 0.001*	< 0.001*
DMN_A	-0.163	< 0.001*	0.054~	-0.149	< 0.001*	0.357
DMN_B	-0.121	0.008*	0.233	-0.161	< 0.001*	0.215
**DMN_C**	**-0.206**	**< 0.001***	**0.006***	**-0.252**	**< 0.001***	**< 0.001***
**DMN_D**	**-0.175**	**0.002***	**0.046***	**-0.193**	**< 0.001***	**0.036***
LIM_A	-0.170	< 0.001*	0.075~	-0.150	< 0.001*	0.380
LIM_B	-0.188	< 0.001*	0.034*	-0.124	0.001*	0.669
**VAN_A**	**-0.188**	**< 0.001***	**0.005***	**-0.173**	**< 0.001***	**0.050***
VAN_B	-0.148	0.003*	0.104	-0.169	< 0.001*	0.128
Visual_A	-0.222	< 0.001*	0.004*	-0.172	< 0.001*	0.205
Visual_B	-0.134	0.005*	0.168	-0.253	< 0.001*	< 0.001*
SMotor_A	-0.098	0.024*	0.270	-0.127	< 0.001*	0.861
SMotor_B	-0.052	0.146	0.895	-0.143	< 0.001*	0.430

r: Pearson's correlation coefficient; *p*: permuted *p*-value (FDR corrected across the 17 networks); *vs.* Control, *p*: Comparison of dependent correlation coefficients (ROI vs. control network/SMotor). * Denotes statistical significance at $p \leq 0.05$; ~ Denotes numeric trend at $p \leq 0.1$. Bolded networks: permuted *p*-value ≤ 0.05 and significantly stronger association correlation than the control network (combined somatomotor A and B, $p \leq 0.05$) in both sexes. Abbreviations: default mode network (DMN), dorsal attention network (DA), ventral attention network (VAN), LIM: limbic, somatomotor (SMotor).

Supplementary Table 4: aHPC-network resting connectivity temporal drift scores

	**Female**			**Male**		
Network	r	p_{FDR}	*vs.* SMotor, *p*	r	p_{FDR}	*vs.* SMotor, *p*
Control_A	-0.110	0.014*	0.737	-0.074	0.023*	0.982
Control_B	-0.075	0.056~	0.893	-0.048	0.094~	0.995
Control_C	-0.020	0.328	0.984	-0.110	0.002*	0.871
DA_A	-0.258	< 0.001*	0.019*	-0.107	0.003*	0.900
DA_B	-0.174	< 0.001*	0.252	-0.168	< 0.001*	0.409
DMN_A	-0.115	0.011*	0.710	-0.090	0.007*	0.926
DMN_B	-0.079	0.056*	0.893	-0.073	0.025*	0.966
DMN_C	-0.085	0.046*	0.849	-0.196	< 0.001*	0.211
DMN_D	-0.193	< 0.001*	0.191	-0.105	0.003*	0.915
LIM_A	-0.213	< 0.001*	0.127	-0.154	< 0.001*	0.555
LIM_B	-0.181	< 0.001*	0.258	-0.047	0.096	0.992
**VAN_A**	**-0.253**	**< 0.001***	**0.007***	**-0.223**	**< 0.001***	**0.030***
VAN_B	-0.169	< 0.001*	0.327	-0.199	< 0.001*	0.177
**Visual_A**	**-0.245**	**< 0.001***	**0.043***	**-0.290**	**< 0.001***	**< 0.001***
Visual_B	-0.159	0.002*	0.410	-0.262	< 0.001*	0.004*
SMotor_A	-0.150	<0.001*	0.424	-0.145	< 0.001*	0.927
SMotor_B	-0.123	0.008*	0.808	-0.138	< 0.001*	0.843

r: Pearson's correlation coefficient; *p*: permuted *p*-value (FDR corrected across the 17 networks); *vs.* Control, *p*: Comparison of dependent correlation coefficients (ROI vs. control network/SMotor). * Denotes statistical significance at $p \leq 0.05$; ~ Denotes numeric trend at $p \leq 0.1$. Bolded networks: permuted *p*-value ≤ 0.05 and significantly stronger correlation than the control network (combined somatomotor A and B, $p \leq 0.05$) in both sexes. Abbreviations: default mode network (DMN), dorsal attention network (DA), ventral attention network (VAN), LIM: limbic, somatomotor (SMotor).

Supplementary Table 5: EC-network resting connectivity temporal drift scores (individualized network parcellation)

	Female			Male		
Network	r	p_{FDR}	*vs.* SMotor, *p*	r	p_{FDR}	*vs.* SMotor, *p*
Control_A	-0.111	0.016*	0.353	-0.269	< 0.001*	< 0.001*
Control_B	-0.148	0.002*	0.131	-0.172	< 0.001*	0.067~
Control_C	-0.080	0.05*	0.577	-0.147	< 0.001*	0.251
**DA_A**	**-0.179**	**0.001***	**0.049***	**-0.200**	**< 0.001***	**0.006***
**DA_B**	**-0.174**	**< 0.001***	**0.020***	**-0.174**	**< 0.001***	**0.033***
DMN_A	-0.156	0.002*	0.105	-0.110	0.002*	0.686
DMN_B	-0.129	0.007*	0.228	-0.179	< 0.001*	0.041
**DMN_C**	**-0.209**	**< 0.001***	**0.009***	**-0.229**	**< 0.001***	**0.002***
DMN_D	-0.163	< 0.001*	0.088~	-0.196	< 0.001*	0.011*
LIM_A	-0.082	0.05 *	0.559	-0.193	< 0.001*	0.037*
LIM_B	-0.241	< 0.001*	0.004*	-0.114	< 0.001*	0.669
**VAN_A**	**-0.156**	**0.002***	**0.026***	**-0.170**	**< 0.001***	**0.028***
VAN_B	-0.178	< 0.001*	0.028*	-0.111	0.001*	0.669
Visual_A	-0.232	< 0.001*	0.004*	-0.113	0.001*	0.614
**Visual_B**	**-0.177**	**< 0.001***	**0.043***	**-0.278**	**< 0.001***	**< 0.001***
SMotor_A	-0.108	0.013*	0.236	-0.143	< 0.001*	0.135
SMotor_B	-0.079	0.053	0.710	-0.141	< 0.001*	0.199

r: Pearson’s correlation coefficient; *p*: permuted *p*-value (FDR corrected across the 17 networks); *vs.* Control, *p*: Comparison of dependent correlation coefficients (ROI vs. control network/SMotor). * Denotes statistical significance at $p \leq 0.05$; ~ Denotes numeric trend at $p \leq 0.1$. Bolded networks: permuted *p*-value ≤ 0.05 and significantly stronger association correlation than the control network (combined somatomotor A and B, $p \leq 0.05$) in both sexes. Abbreviations: default mode network (DMN), dorsal attention network (DA), ventral attention network (VAN), LIM: limbic, somatomotor (SMotor).

Supplementary Table 6: aHPC-network resting connectivity temporal drift scores (individualized network parcellation)

	Female			Male		
Network	r	p_{FDR}	*vs.* SMotor, *p*	r	p_{FDR}	*vs.* SMotor, *p*
Control_A	-0.148	0.002*	0.549	-0.111	0.003*	0.884
Control_B	-0.031	0.280	0.985	-0.021	0.286	0.997
Control_C	-0.002	0.499	0.996	-0.041	0.138	0.991
DA_A	-0.227	< 0.001*	0.090~	-0.090	0.008*	0.929
DA_B	**-0.274**	**< 0.001***	**0.003***	**-0.230**	**< 0.001***	**0.027***
DMN_A	-0.142	0.002*	0.595	-0.061	0.064	0.970
DMN_B	-0.076	0.066~	0.924	-0.040	0.138	0.989
DMN_C	-0.106	0.020*	0.807	-0.158	< 0.001*	0.507
DMN_D	-0.210	< 0.001*	0.163	-0.140	< 0.001*	0.663
LIM_A	-0.082	0.053~	0.878	-0.167	< 0.001*	0.423
LIM_B	-0.290	< 0.001*	0.007*	-0.050	0.101*	0.989
VAN_A	-0.209	< 0.001*	0.090~	-0.302	< 0.001*	< 0.001*
VAN_B	-0.187	< 0.001*	0.256	-0.181	< 0.001*	0.309
Visual_A	-0.209	< 0.001*	0.178	-0.194	< 0.001*	0.174
Visual_B	-0.235	< 0.001*	0.083~	-0.314	< 0.001*	< 0.001*
SMotor_A	-0.159	0.001	0.428	-0.155	< 0.001*	0.607
SMotor_B	-0.133	0.007	0.803	-0.154	< 0.001*	0.589

r: Pearson's correlation coefficient; *p*: permuted *p*-value (FDR corrected across the 17 networks); *vs.* Control, *p*: Comparison of dependent correlation coefficients (ROI vs. control network/SMotor). * Denotes statistical significance at $p \leq 0.05$; ~ Denotes numeric trend at $p \leq 0.1$. Bolded networks: permuted *p*-value ≤ 0.05 and significantly stronger correlation than the

control network (combined somatomotor A and B, $p \leq 0.05$) in both sexes. Abbreviations: default mode network (DMN), dorsal attention network (DA), ventral attention network (VAN), LIM: limbic, somatomotor (SMotor).

Supplementary References:

1. Zhang, Y., Brady, M. & Smith, S. Segmentation of brain MR images through a hidden Markov random field model and the expectation-maximization algorithm. *IEEE Trans. Med. Imaging* **20**, 45–57 (2001).
2. Tambini, A., Rimmele, U., Phelps, E. A. & Davachi, L. Emotional brain states carry over and enhance future memory formation. **20**, (2017).
3. Zou, F. *et al.* Re-expression of CA1 and entorhinal activity patterns preserves temporal context memory at long timescales. *Nat. Commun.* **14**, 4350 (2023).
4. Radvansky, G. A. & Zacks, J. M. Event Boundaries in Memory and Cognition. *Curr Opin Behav Sci* **17**, 133–140 (2017).
5. Eichenbaum, H. On the Integration of Space, Time, and Memory. *Neuron* **95**, 1007–1018 (2017).
6. Bellmund, J. L. S., Polti, I. & Doeller, C. F. Sequence memory in the hippocampal-entorhinal region. *J. Cogn. Neurosci.* **32**, 2056–2070 (2020).
7. Clewett, D., DuBrow, S. & Davachi, L. Transcending time in the brain : How event memories are constructed from experience. *Hippocampus* 1–22 (2019).
8. Sugar, J. & Moser, M.-B. Episodic memory: Neuronal codes for what, where, and when. *Hippocampus* **29**, 1190–1205 (2019).
9. Buzsáki, G. & Tingley, D. Space and Time: The Hippocampus as a Sequence Generator. *Trends Cogn. Sci.* **22**, 853–869 (2018).
10. Palombo, D. J. & Cocquyt, C. Emotion in Context : Remembering When. *Trends Cogn. Sci.* **24**, 687–690 (2020).
11. Wang, J., Tambini, A. & Lapate, R. C. The tie that binds: temporal coding and adaptive emotion. *Trends Cogn. Sci.* (2022) doi:10.1016/j.tics.2022.09.005.

12. Bright, I. M. *et al.* A temporal record of the past with a spectrum of time constants in the monkey entorhinal cortex. *Proceedings of the National Academy of Sciences* **117**, 20274–20283 (2020).
13. Howard, M. W. Memory as Perception of the Past: Compressed Time in Mind and Brain. *Trends Cogn. Sci.* **22**, 124–136 (2018).
14. Kong, R. *et al.* Individual-specific areal-level parcellations improve functional connectivity prediction of behavior. *Cereb. Cortex* **31**, 4477–4500 (2021).
15. Kong, R. *et al.* Spatial Topography of Individual-Specific Cortical Networks Predicts Human Cognition, Personality, and Emotion. *Cereb. Cortex* **29**, 2533–2551 (2019).
