# Supplementary material for: The intrinsic time tracker: temporal context is embedded in entorhinal and hippocampal functional connectivity patterns
Source: Nat Commun. 2025 Oct 3;16:8817. doi: 10.1038/s41467-025-63633-6 (PMC12494713; doi:10.1038/s41467-025-63633-6)
Supplement: Supplementary file 2 — Reporting Summary [file 41467_2025_63633_MOESM2_ESM.pdf]

nature portfolio

Corresponding author(s): Regina C Lapate

Last updated by author(s): Jul 29, 2025

Reporting Summary

Nature Portfolio wishes to improve the reproducibility of the work that we publish. This form provides structure for consistency and transparency in reporting. For further information on Nature Portfolio policies, see our Editorial Policies and the Editorial Policy Checklist.

Statistics

For all statistical analyses, confirm that the following items are present in the figure legend, table legend, main text, or Methods section.

n/a	Confirmed	
☐	☒	The exact sample size (*n*) for each experimental group/condition, given as a discrete number and unit of measurement
☐	☒	A statement on whether measurements were taken from distinct samples or whether the same sample was measured repeatedly
☐	☒	The statistical test(s) used AND whether they are one- or two-sided<br>*Only common tests should be described solely by name; describe more complex techniques in the Methods section.*
☐	☒	A description of all covariates tested
☐	☒	A description of any assumptions or corrections, such as tests of normality and adjustment for multiple comparisons
☐	☒	A full description of the statistical parameters including central tendency (e.g. means) or other basic estimates (e.g. regression coefficient) AND variation (e.g. standard deviation) or associated estimates of uncertainty (e.g. confidence intervals)
☐	☒	For null hypothesis testing, the test statistic (e.g. *F*, *t*, *r*) with confidence intervals, effect sizes, degrees of freedom and *P* value noted<br>*Give P values as exact values whenever suitable.*
☒	☐	For Bayesian analysis, information on the choice of priors and Markov chain Monte Carlo settings
☒	☐	For hierarchical and complex designs, identification of the appropriate level for tests and full reporting of outcomes
☐	☒	Estimates of effect sizes (e.g. Cohen's *d*, Pearson's *r*), indicating how they were calculated

Our web collection on statistics for biologists contains articles on many of the points above.

Software and code

Policy information about availability of computer code

Data collection	The female subject dataset is openly available at https://openneuro.org/datasets/ds002674. The male subject dataset is openly available at https://openneuro.org/datasets/ds005115.
Data analysis	Analyses were run using custom code in FSL (version 6.0.7.12), Python (version 3.8, Package: Nilearn_0.9.1), AFNI (Version 24.1.22), and R (R studio Version 1.4.1717, R version 4.1.2. Packages: tidyr_1.2.1; dplyr_1.0.10; emmeans_1.6.3; stats_4.1.2; cocor_1.1.3; ggplot2_3.4.2), which is available in GitHub repository (https://github.com/LEAPNeuroLab/rsFCTemporalDrift).

For manuscripts utilizing custom algorithms or software that are central to the research but not yet described in published literature, software must be made available to editors and reviewers. We strongly encourage code deposition in a community repository (e.g. GitHub). See the Nature Portfolio guidelines for submitting code & software for further information.

Data

Policy information about availability of data

All manuscripts must include a data availability statement. This statement should provide the following information, where applicable:

- Accession codes, unique identifiers, or web links for publicly available datasets
- A description of any restrictions on data availability
- For clinical datasets or third party data, please ensure that the statement adheres to our policy

The female subject dataset is openly available at https://openneuro.org/datasets/ds002674. The male subject dataset is openly available at https://openneuro.org/datasets/ds005115.

Research involving human participants, their data, or biological material

Policy information about studies with human participants or human data. See also policy information about sex, gender (identity/presentation), and sexual orientation and race, ethnicity and racism.

Reporting on sex and gender	Two healthy, right-handed adults (1 Female, 23 years; 1 Male, 26 years) with normal or corrected-to-normal vision participated. Both participants provided consent.
Reporting on race, ethnicity, or other socially relevant groupings	Both participants were Caucasian.
Population characteristics	Two healthy adults (1 Female, 23 years; 1 Male, 26 years).
Recruitment	Participants were recruited from the University of California, Santa Barbara, CA.
Ethics oversight	The study was approved by the University of California, Santa Barbara Human Subjects Committee.

Note that full information on the approval of the study protocol must also be provided in the manuscript.

Field-specific reporting

Please select the one below that is the best fit for your research. If you are not sure, read the appropriate sections before making your selection.

☒ Life sciences ☐ Behavioural & social sciences ☐ Ecological, evolutionary & environmental sciences

For a reference copy of the document with all sections, see nature.com/documents/nr-reporting-summary-flat.pdf

Life sciences study design

All studies must disclose on these points even when the disclosure is negative.

Sample size	This dense sampling dataset contained two participants.
Data exclusions	Overall motion was negligible (mean framewise displacement < 0.4) so all the fMRI sessions were included in the current study. Between-session similarity values were considered outliers and excluded from subsequent analyses if they exceeded 3 standard deviations from the mean across all pairs of sessions for each seed region [fewer than 1% session-pairs were excluded for each seed region, range of session pairs included: Female: 432 - 435 sessions (Mean = 434.087, SD = 0.848); Male: 769-780 sessions (Mean = 778.783, SD = 2.522)].
Replication	N/A
Randomization	We controlled time-varying factors [including motion (mean FD), hormone fluctuations, and mood state changes] when using time intervals to predict changes in resting connectivity similarity between session pairs.
Blinding	N/A

Reporting for specific materials, systems and methods

We require information from authors about some types of materials, experimental systems and methods used in many studies. Here, indicate whether each material, system or method listed is relevant to your study. If you are not sure if a list item applies to your research, read the appropriate section before selecting a response.

Materials & experimental systems

n/a	Involved in the study
☒	☐ Antibodies
☒	☐ Eukaryotic cell lines
☒	☐ Palaeontology and archaeology
☒	☐ Animals and other organisms
☒	☐ Clinical data
☒	☐ Dual use research of concern
☒	☐ Plants

Methods

n/a	Involved in the study
☒	☐ ChIP-seq
☒	☐ Flow cytometry
☐	☒ MRI-based neuroimaging

Plants

Seed stocks	N/A
Novel plant genotypes	N/A
Authentication	N/A

Magnetic resonance imaging

Experimental design

Design type	Resting-state fMRI data and high-resolution T1- and T2-weighted images were used. Hormone and questionnaire results were also used.
Design specifications	The female subject underwent one session per day for 30 consecutive days with each session starting at 11:00 AM. The male subject also underwent 30 days of repeated testing, with the first 10 days at 7:00 AM, the second 10 days at 7:00 AM and 8:00 PM, and the final third 10 days at 8:00 PM. Serum and salivary assessments of hormones (Female: estradiol, progesterone, luteinizing hormone (LH) and follicle-stimulating hormone (FSH); Male: estradiol, testosterone, cortisol) and questionnaires (Perceived Stress Scale, State-Trait Anxiety Inventory for Adults, and Profile of Mood States) were collected at the start of each session. Participants then completed T1- and T2-weighted structural scans, followed by T2*-weighted resting-state fMRI scans with their eyes open (Female: 10 minutes, Male: 15 minutes).
Behavioral performance measures	No behavioral measures were acquired during the fMRI scan recordings.

Acquisition

Imaging type(s)	Functional & Structural
Field strength	3T
Sequence & imaging parameters	T1-weighted whole-brain structure scan: repetition time [TR] = 720 ms; echo time [TE] = 37 ms; 2.0-mm isotropic voxels, multiband factor = 8. T2-weighted hippocampal scan: acquired with an oblique coronal orientation positioned orthogonally to the main axis of the hippocampus; TR = 8100ms; TE = 8100, 0.4 X 0.4 mm2 in-plane resolution; 2 mm slice thickness. T2*-weighted resting-state fMRI scan: TR = 720 ms; TE = 37 ms; 2.0-mm isotropic voxels, multiband factor = 8.
Area of acquisition	T1-weighted and T2*-weighted resting-state fMRI scans are whole brain scans. T2-weighted scans were focused on the hippocampus.

Diffusion MRI ☐ Used ☒ Not used

Preprocessing

Preprocessing software	FSL (version 6.0.7.12), Python (version 3.8, Package: Nilearn_0.9.1), and R (R studio Version 1.4.1717, R version 4.1.2. Packages: tidyr_1.2.1; dplyr_1.0.10; emmeans_1.6.3; stats_4.1.2; cocor_1.1.3; ggplot2_3.4.2)
Normalization	Functional images were co-registered to the participants' T1-weighted anatomical image using a linear rigid body (6-DOF) transform while maintaining native functional resolution (2 mm3 isotropic).

Normalization template	Analyses were conducted on participants' structural space (see normalization above).
Noise and artifact removal	Six motion parameters and the average signal obtained from FSL-derived cerebrospinal fluid and white matter masks (obtained using FAST) were entered as nuisance regressors using AFNI's 3dDeconvolve function. Next, a band-pass filter (0.01 Hz < f < 0.1 Hz) was applied using AFNI's 3dBandpass function.<br><br>Outliers of between-session similarity values were excluded from subsequent analyses if they exceeded 3 standard deviations from the mean across all pairs of sessions for each seed region (fewer than 1% session-pairs were excluded for each seed region, range of session pairs included: Female: 432 - 435 sessions (Mean = 434.087, SD = 0.848); Male: 769-780 sessions (Mean = 778.783, SD = 2.522)).
Volume censoring	N/A

Statistical modeling & inference

Model type and settings	Regional-whole brain temporal drift score:<br>To obtain regional-whole brain resting-state connectivity patterns for each session, we first averaged the time series across all voxels within each region of interest (ROI). To measure the connectivity pattern in each session, Pearson's correlation values (indexing resting-state functional connectivity with each seed) were obtained using AFNI for all gray-matter voxels, which were Fisher-z transformed. Next, we calculated Pearson's correlation coefficients (i.e., indexing similarity) for resting-state functional connectivity patterns between every pair of sessions (across all gray matter voxels), which were Fisher-z-transformed. Finally, to capture whether connectivity patterns reliably tracked elapsed time, a temporal drift score was calculated for each seed ROI. To do this, we correlated the similarity of connectivity patterns (Z-transformed correlation coefficients obtained for every session pair) with the Δ time interval between session pairs.<br><br>Regional-cortical network temporal drift scores:<br>To isolate whether relationships with elapsed time were specific to particular large-scale cortical networks, temporal drift scores were calculated in connectivity patterns of established large-scale cortical networks. Temporal drift scores for the entorhinal cortex and anterior hippocampus regions were calculated as described above (for the whole brain), now with each of the Yeo-17 networks.<br><br>Hippocampus longitudinal axis analysis:<br>Temporal drift scores for the analysis of the hippocampus longitudinal axis were calculated as described above, but using single-voxels in the hippocampus as seeds instead of the average time series in the ROI.<br><br>Entorhinal cortex subregional analysis:<br>Voxel-wise temporal drift scores in the EC were calculated and then averaged within each EC subregion (i.e., anterolateral and posteromedial entorhinal cortex.
Effect(s) tested	We tested whether the strength of the entorhinal and anterior hippocampal whole-brain resting-state functional connectivity patterns became increasingly dissimilar as time elapsed, and whether these time-dependent changes were specific to specific large-scale cortical networks. Moreover, we tested whether the strength of this intrinsic time tracking varied systematically along the hippocampus and subregions in the entorhinal cortex.

Specify type of analysis: ☐ Whole brain ☒ ROI-based ☐ Both

Anatomical location(s)	Hippocampus, entorhinal cortex, perirhinal cortex, primary motor cortex, and cortical networks derived from Yeo-17 MNI atlas.

Statistic type for inference<br><br>(See Eklund et al. 2016)	We parcellated the medial temporal lobe (MTL) into the hippocampus, entorhinal cortex, and perirhinal cortex using the Automatic Segmentation of Hippocampal Subfields package (ASHS) (Taylor et al., 2020). Then, a trained neuroanatomist (Dr. Jingyi Wang) manually inspected (and when needed, corrected) the ASHS masks. The hippocampus was divided into anterior and posterior regions based on the presence of uncus (Strange et al., 2014). The entorhinal cortex was divided into anterolateral and posteromedial subregions using parcellation strategy published in Maass et al., 2015. M1 (primary motor cortex) was obtained from the Oxford PFC Consensus Atlas. We also used the Yeo-17 MNI atlas (Yeo et al., 2011) and individual network parcellation strategy (Kong et al., 2019) for the network analysis
Correction	For network analysis, results were FDR corrected across the 17 Yeo network ROIs.

Models & analysis

n/a	Involved in the study
☐	☒ Functional and/or effective connectivity
☒	☐ Graph analysis
☐	☒ Multivariate modeling or predictive analysis

Functional and/or effective connectivity	To measure the connectivity pattern for each ROI in each session, Pearson's correlation values (indexing resting-state functional connectivity with each seed) were obtained using AFNI for all gray-matter voxels, which were Fisher-z transformed (for details see above).
Multivariate modeling and predictive analysis	N/A
